# Characteristics of *Serratia rubidaea* Clinical Strain Revealed Multiple Resistance to Antibiotics and Disinfectants

**DOI:** 10.3390/microorganisms14050988

**Published:** 2026-04-28

**Authors:** Anfisa Kozyreva, Anna Akhmetzyanova, Alexey Kovalenko, Ivan Chudinov, Irina Rog, Elena Korneenko, Anastasia Vakaryuk, Veronica Gremyacheva, Ivan Butenko, Vadim Govorun

**Affiliations:** Scientific Research Institute for Systems Biology and Medicine, Federal Service on Consumer Rights Protection and Human Well-Being Surveillance, Moscow 117246, Russia; anfisakozyr@gmail.com (A.K.); ahmetzyanova_aa@sysbiomed.ru (A.A.);

**Keywords:** multiomics, *Serratia* spp., bacteriological surveillance, HPLC/MS-LC, efflux pumps, WGS, antibiotic susceptibility testing, *Serratia rubidaea*, MDR, antimicrobial resistance

## Abstract

A clinical strain of the opportunistic pathogen *Serratia rubidaea*, a known contaminant of healthcare environments and an emerging cause of invasive infections, is described. The studied isolate, recovered from a nurse’s hand skin swab during routine screening, exhibits a broad profile of antibiotic resistance combined with reduced susceptibility to several disinfectants. Phenotypic susceptibility testing using a tablet-based microdilution and disk diffusion method was employed to determine the minimum inhibitory concentrations (MICs) of antimicrobial agents from different classes, while broth microdilution assays with disinfectants revealed high-level tolerance to widely used agents, including 70% C_2_H_5_OH, 3% H_2_O_2_, 0.05% polyhexamethylene guanidine (PHMG) and others. Whole-genome sequencing identified multiple resistance-associated determinants, such as chromosome-encoded class C β-lactamase (*ampC*), several efflux systems (*sdeXY*, *macAB*, and *emrAB*) combined with multicopy *tolC*, and specific transferases (*fos* and *arnT*). Shotgun bottom-up HPLC-MS/MS proteomics confirmed baseline expression of these and other stress-tolerance-related proteins under non-inducing conditions. Taken together, these data underscore the importance of surveillance for *Serratia* spp. in healthcare facilities to detect strains that combine intrinsic or acquired multidrug resistance with robust survival traits such as disinfectant tolerance and biofilm formation. The present study provides a reference-level phenotypic, genomic, and proteomic characterization of a *S. rubidaea* clinical isolate, contributing to the understanding of the adaptive potential of this resilient opportunistic pathogen in clinical environments.

## 1. Introduction

Over the past few decades, the global spread of antibiotic resistance among bacteria has become a major public health concern. The contamination of medical equipment and hospital environments with opportunistic pathogens that combine broad intrinsic resistance with adaptive and acquired multidrug resistance can substantially complicate the clinical management of infections [1,2,3]. Antibiotic resistance reduces the effectiveness of treatment and can lead to severe complications and increased mortality, especially when initial therapy is inappropriate [4]. The treatment of infections caused by multidrug-resistant agents puts a severe economic burden on healthcare systems around the world [5]. These factors highlight the importance of regular resistance surveillance, including systematic screening of clinical and environmental isolates in hospital settings where the risk of the dissemination of resistant strains is particularly high.

Within the order Enterobacterales, the genus *Serratia* (class Gammaproteobacteria, family Enterobacteriaceae) comprises opportunistic Gram-negative bacteria that are frequently recovered from diverse ecological niches, such as plants, soil, water, and food, as well as from healthcare environments. *Serratia* spp. are characterized by a broad spectrum of intrinsic antibiotic resistance and a notable capacity to acquire additional resistance determinants. For various *Serratia* species, resistance has been reported to multiple clinically important classes of antimicrobials, including β-lactams, aminoglycosides, fluoroquinolones, fosfomycin, tetracyclines, and others that often represent first-line or key therapeutic options [6]. In addition, many *Serratia* isolates have been shown to form robust biofilms on abiotic surfaces, which is consistent with their frequent detection on medical devices and equipment and may contribute to persistent contamination and difficult-to-treat infections, especially in immunocompromised patients or those with disrupted normal microbiota [7,8,9,10]. Among these organisms, *Serratia rubidaea* has long been regarded primarily as an environmental species and a contaminant of healthcare facilities. However, its clinical relevance is increasingly being recognized as an agent of invasive infection. Case reports and small series have described episodes of *S. rubidaea* bacteremia in intensive care units and other hospital wards, underscoring its potential to act as an opportunistic pathogen rather than a mere contaminant [8,10]. Despite these observations, systematic data on the resistance mechanisms, disinfectant tolerance, and genomic features of *S. rubidaea* isolates from hospital environments remain scarce. Most available studies either focus on single clinical cases without in-depth molecular characterization or address the most common member of *Serratia* spp.—*Serratia marcescens* [6,7,9].

In this context, the characterization of *S. rubidaea* isolates recovered from hospital environments, particularly from the hands of healthcare workers, is important due to several aspects. First, such isolates may serve as reservoirs of intrinsic and acquired resistance determinants that can complicate treatment if invasive infection occurs. Second, tolerance to commonly used disinfectants and the ability to persist on medical surfaces or skin may facilitate the silent circulation of these bacteria in healthcare facilities. Third, there is currently a lack of integrated phenotypic, genomic, and proteomic data for *S. rubidaea*, which limits understandings of its adaptive strategies and the potential risks associated with its presence in clinical settings.

The present study provides a comprehensive characterization of a *S. rubidaea* isolate (strain no. 151, SERRU_NIISBM) recovered from a nurse’s hand swab during a routine microbiological screening in a healthcare facility. The study aims to determine the antimicrobial susceptibility profile of this strain, assess its tolerance to a set of disinfectants and antiseptics that are widely used in Russian medical practice, and identify determinants potentially underlying antibiotic resistance, disinfectant tolerance, and other stress-related traits. By linking phenotypic resistance and disinfectant tolerance with genome-encoded and proteomically confirmed mechanisms, this work aims to address the current gap in molecular data on *S. rubidaea* from hospital environments and provides a reference description of a clinically relevant isolate that may inform infection control and empirical therapy considerations.

## 2. Materials and Methods

### 2.1. Sample Collection

The studied isolate was collected as part of the routine microbiological control procedures for medical care organizations, hospitals, and medical laboratories, as established by the Methodological Recommendations MR 3.1.0346-24 “Organization and Conduct of Microbiological Monitoring in Medical Organizations” [11] (approved by Federal Service for the Oversight of Consumer Protection and Welfare of the Russian Federation on 26 April 2024). The studied strain was isolated in 2018 from a hand skin swab of a nurse at the S.P. Botkin State Clinical Hospital, Moscow, Russia. For some comparative analysis, genomes of closely related strains from NCBI RefSeq were retrieved and mentioned as a “Reference set“; details are indicated in Section 2.9.

### 2.2. Species Identification

The studied strain was identified as *Serratia rubidaea* using the VITEK® MS IVD (bioMérieux, Marcy-l’Étoile, France) and DL Smart MS 5020 (Zhuhai DL Biotech Co., Zhuhai, China) automated systems based on time-of-flight mass spectrometry. Reclassification of pure culture during the study was performed using a DL Smart MS 5020 time-of-flight mass spectrometer (Zhuhai DL Biotech Co.), integrated with an extensive microorganism database using DL Mass Software, V1.1.1.0, and an MS-7-ZOOM stereoscopic trinocular microscope (Micromed, Taipei, Taiwan). The DL Smart MS 5020 mass spectrometer was calibrated using the purified protein extract of *Escherichia coli*—the “ALMASS Bio Standard” standard (Algimed Techno, Moscow, Russia), and the samples were prepared using “ALMASS Bio” reagents (Algimed Techno) in accordance with the manufacturer’s instructions.

### 2.3. Microbiology

Cultivation was performed using a nutrient broth based on fish meal hydrolysate (GRM) (FBUN SRCAMB, Obolensk, Russia), with an inoculum dose of 100 μL added to 9 mL of the medium. The cultures were incubated for 24 h on a shaker at 180 rpm and a temperature of 37±1 °C under aerobic conditions. The optical density of the resulting bacterial suspension was $9\times 10^{8}$ CFU/mL, corresponding to a 3.0 McFarland turbidity standard. To assess morphology, the obtained culture was plated onto a solid differential-selective medium—Endo agar (FBUN SRCAMB)—using the Gold streak method to achieve isolated colonies. Petri dishes were incubated at 37±1 °C in an incubator for 24 h. Subsequently, the morphological properties of individual colonies were evaluated after Gram staining using a BIOLOGICAL MICROSCOPE MT 4200 L optical microscope (Ohaus Instruments, Shanghai, China). An extended description of the morphological properties of the pure studied culture is placed in Supplementary File, Section S2.

### 2.4. Antibiotic Resistance Assessment

Antimicrobial susceptibility testing was performed using commercial MICROLATEST MIC G-I and MIC G-II panels (Erba Mannheim, Brno, Czech Republic) and the disk diffusion method (NITsF, Saint Petersburg, Russia). The antimicrobial panel included 24 drugs from the following groups: β-lactams, aminoglycosides, tetracyclines, sulfonamides, fluoroquinolones, polymyxins, as well as chloramphenicol and trimethoprim. Results were interpreted according to EUCAST (European Committee on Antimicrobial Susceptibility Testing) for the Enterobacterales or CLSI (The Clinical & Laboratory Standards Institute) criteria (for cefazolin, tetracycline, and cefoperazone). The whole list of the tested antibiotics and their concentrations can be found in Supplementary File, Section S3.

The antibacterial agents susceptibility testing procedure comprised the following steps.

1. Broth Microdilution Method [12]: A working suspension was prepared from a 24 h culture using Mueller–Hinton broth (HiMedia) and saline. The final bacterial suspension concentration was adjusted to 0.5 McFarland standard. Subsequently, 60 μL of the suspension was inoculated into 13 mL of sterile Mueller–Hinton broth. The resulting inoculum (100 μL) was dispensed into the plate wells, which were sealed with a film and incubated at 37±1 °C. The results were recorded after incubation according to the manufacturer’s instructions;
2. Disk Diffusion Method [12]: For the disk diffusion assay using antibiotic discs (for erythromycin, norfloxacin, and fosfomycin), the working suspension was plated onto Mueller–Hinton agar (HiMedia) plates using the lawn method. Antibiotic discs were then aseptically placed on the agar surface. Incubation and result interpretation were performed in accordance with the relevant methodological guidelines [12,13].

### 2.5. Disinfectants Tolerance Assessment

The biocidal activity of hydrogen peroxide (H_2_O_2_), alkyldimethylbenzylammonium chloride (ADBAC), polyhexamethylene guanidine (PHMG), a tertiary amine (Triam.), sodium dichloroisocyanurate (NaDCC), glutaraldehyde, and ethanol (C_2_H_5_OH) was assessed against the studied strain. The assays were performed in 96-well plates using a neutralization method followed by plating onto solid GRM agar with inoculation into GRM broth, in accordance with the national guidelines [12,13] and CDC “Guideline for disinfection and sterilization in healthcare facilities” [14]. Each experiment was performed in triplicate. Each sample was tested at three different concentrations of each disinfectant. The results were recorded after 24 h of incubation at 37±1 °C.

The procedure for performing the disinfectant tolerance assessment included the following.

1. Preparation of the bacterial suspension: A pure culture of the studied strain was grown on a solid medium for 24 h. Then, it was suspended in sterile saline solution to achieve an optical density corresponding to $10^{8}$ CFU/mL;
2. Preparation of disinfectant solutions: Each disinfectant was prepared according to the manufacturer’s instructions and diluted to the required concentrations using sterile distilled water;
3. Neutralization method: The bacterial suspension (100 μL) was added to each well of a 96-well plate, followed by the addition of 100 μL of the disinfectant solution. After incubation for 5 min at room temperature, 800 μL of neutralizing broth was added to each well to stop the biocidal action;
4. Plating onto solid GRM agar: From each well, 100 μL of the mixture was plated onto a solid GRM agar plate and spread evenly using a sterile spreader. The plates were incubated at 37±1 °C for 24 h.

### 2.6. Nucleic Acids and Proteins Extraction

The pure bacterial culture cell pellet was resuspended in 500 μL of 2% CTAB buffer (2% CTAB, 1.4 M NaCl, 0.1 M Tris-HCl, and 20 mM EDTA) and incubated at 65±1 °C for 30 min. Then, 500 μL of chloroform was added, and the mixture was vortexed and centrifuged at 21,000× *g* for 20 min at 4±1 °C. The upper aqueous phase was collected and treated with RNase A (Biolabmix, Novosibirsk, Russia) at a concentration of 1 μL per 100 μL of supernatant, followed by incubation for 5 min at room temperature. Subsequently, 1/10 volume of 3 M sodium acetate (pH 5.2) and 1 volume of isopropanol were added, and the mixture was incubated for 1 h at −20±1 °C. After incubation, the sample was centrifuged at 21,000× *g* for 40 min at 4±1 °C. The isopropanol was carefully removed without disturbing the pellet, which was then washed with 100 μL of 80% ethanol and centrifuged again at 21,000× *g* for 10 min at 4±1 °C. The ethanol was removed, and the pellet was air-dried for 5 min at room temperature with the tube open. The DNA was resuspended in 25 μL of TE buffer (10 mM Tris-HCl and 1 mM EDTA) and incubated at 37±1 °C for 30 min. The extracted DNA was further purified using MGI Easy DNA Clean Beads (MGI, Shenzhen, China) at a 0.8:1 ratio (beads to sample). To obtain the cell pellet and supernatant, a pure bacterial culture of the studied strain was cultivated under conditions identical to those described in the Section 2.3. The resulting culture was centrifuged at 3000× *g* for 20 min at 4±1 °C. Protein fraction was isolated from the cell pellet by protein precipitation using organic solvents (chloroform–methanol). The resulting protein mixtures in solution were treated identically with Trypsin Gold (Promega, Madison, WI, USA) for proteolytic digestion. The resulting mixture of tryptic peptides was used for chromatographic-mass spectrometric proteomic analysis.

### 2.7. Whole-Genome Sequencing

Whole-genome sequencing was carried out using both second- and third-generation sequencing technologies on the MGI DNBSEQ-G400 (MGI) and Oxford Nanopore Technologies PromethION 24 (Oxford Nanopore Technologies (ONT), Oxford, UK) platforms. All library-preparing steps were made according to the manufacturers’ standard protocols. For ONT-sequencing libraries were prepared using the Rapid sequencing DNA V14-barcoding protocol with the SQK-RBK114.96 kit (ONT) and sequenced on a FLO-PRO-114M flow cell. For MGI-sequencing, libraries were prepared with the following kits and components: MGIEasy FS DNA Library Prep Kit, MGIEasy DNA Adapters-96 (Plate) Kit, MGIEasy DNA Clean Beads, MGIEasy Circularization Module (MGI), and a DNBSEQ-G400RS FCS PE150 cartridge and flow cell (MGI).

### 2.8. Shotgun Bottom-Up HPLC-MS/MS

HPLC-MS analysis of the obtained peptide samples was performed using an Orbitrap Exploris 480 mass spectrometer (Thermo Scientific, Waltham, MA, USA) coupled with an Ultimate 3000 RSLCnano chromatographic system (Thermo Scientific) via a Nanospray Flex ion source (Thermo Scientific). The samples were dissolved in 35 μL of 5% acetonitrile (HPLC Grade) in water (LC-MS Grade) with 0.1% formic acid (*v*/*v*). The chromatographic separation of peptides was performed by reversed-phase chromatography using a C18 PepMap 100 precolumn (sorbent—C18, length 5 mm, internal diameter 300 μm, particle size 5 μm, and pore diameter 100 A, Thermo Scientific, USA) and a Peaky-C18 capillary column (sorbent—C18, length 50 cm, internal diameter 75 μm, and particle size 1.9 μm, Molekta, Russia). Each sample in a volume of 5 μL was applied to the precolumn in 5% acetonitrile (HPLC Grade) in water (LC-MS Grade) with 0.1% formic acid (*v*/*v*) at a flow rate of 10 μL/min for 12 min, after which the precolumn was included in the line before the column. Peptides were eluted in two different gradient variants with a mixture of solvents A (5% acetonitrile (HPLC grade) in water (LC-MS grade) with 0.1% formic acid (*v*/*v*)) and B (79.9% acetonitrile (HPLC grade) (*v*/*v*), 20% water (LC-MS grade) and 0.1% formic acid (*v*/*v*)), increasing the content of solvent B from 5% to 10% over 3 min (12 min for long gradient variant), then from 10% to 55% over 54 min (216 min for long gradient variant), and from 55% to 65% (*v*/*v*) over 3 min (12 min for long gradient variant) at a flow rate of 250 nL/min, after which the system was washed for 2 min with a mixture of 100% (*v*/*v*) solvent B and then for 15 min with a mixture of 5% solvent B. The source voltage was 2200 V, and the capillary temperature was 275 °C. The mass spectrometer operated in the data-dependent analysis mode: in each cycle lasting 1 second, a survey mass spectrum was recorded, and ions were automatically selected from it for the fragmentation and recording of the MS2 spectrum. Parent ions for which a fragmentation spectrum had already been obtained were excluded from consideration automatically. The overview mass spectrum was recorded at a resolution of 60,000 in the mass-to-charge range from 200 to 1500 *m*/*z* with the automatic gain control (AGC) parameter “Standard” and the fill time limit “Auto”. The Funnel RF Level was set to 50. The daughter ion spectra were recorded at a resolution of 15,000 and the AGC-“Standard” with the fill time limit “Auto”. The collision energy parameter was equal to 30, the bandwidth was 1.4 *m*/*z*.

### 2.9. Bioinformatic Analysis

Reads quality control was performed using FastQC v0.12.1 [15] and Nanoplot v1.42.0 [16]. A hybrid assembly was generated from both sequencing technologies using Unicycler v0.5.1 [17] in conservative mode. The assembly was reoriented with dnaapler v1.2.0 [18] to start with the *dnaA* (chromosome) and *repA* (plasmid) genes. Completeness was assessed with BUSCO v5.6.1 against the enterobacterales_odb10 database [19]. Genome annotation was performed using Prokka v1.14.6 [20] and the NCBI Prokaryotic Genome Annotation Pipeline (PGAP) v.2024-07-18.build7555 [21,22]. Antimicrobial resistance (AMR) genes were identified using NCBI AMRfinder v3.12.8 [23] and the Comprehensive Antibiotic Resistance Database (CARD v3.2.7) [24] with the RGI tool. The nearest reference organism was identified using KmerFinder [25]. Multi-Locus Sequence Typing (MLST) was performed with mlst v2.23.0 [26] and the PubMLST database [27]. For phylogenetic analysis, the genomes of closely related strains were retrieved from NCBI RefSeq and mentioned as “Reference set”. Details about th esequences retrieved as the “Reference set” are indicated in Table 1. A pangenome analysis of the “Reference set” strains with the target studied strain was conducted using the Prokka annotations with Panaroo v1.5.2 [28], and a phylogenetic tree was inferred with IQ-TREE2 v2.0 [29] with 1000 replicates bootstrap analysis and visualized in ITOL v7.2 [30].

All proteomic data acquired by shotgun bottom-up HPLC-MS/MS were processed using FragPipe [31]. For the comparative analysis of protein expression, label-free quantification was performed using the intensity-based absolute quantification (iBAQ) metric, which allows approximate comparison of protein abundances within a single sample by normalizing peptide intensities to the number of theoretically observable peptides for each protein. This approach has been widely applied for global and targeted proteomic studies as a robust semi-quantitative indicator of relative protein levels. Proteomic samples were obtained from cultures grown under non-stress conditions in a nutrient-rich medium, without exposure to antibiotics or disinfectants, in order to capture the basal expression profile. Based on the whole-genome analysis of the studied strain, two gene sets were defined: a housekeeping (HK) group and a target group. The HK group included genes encoding stable core cellular functions (*rpoB*, *gyrA*, *gyrB*, *dnaN*, and *mreB*) and was used to establish a reference baseline of constitutive protein abundance. The target group comprised proteins of genes associated with antibiotic resistance, efflux, stress tolerance, and transport: *ompA*, *robA*, *rsmA*, *ompF*, *crp*, *arsD*, *sdeX*, *sdeY*, *acrR*, *acr*-cluster, *mprA*, *tolC*, *msbA*, *ompC*, *macA*, *ompX*, *emrA*, *ompW*, *glpT*, *h-ns*, *macB*, *ampC*, *arsC*, and *fos*. For each protein, log10-transformed iBAQ values were calculated, and the median of the HK group was used as an internal reference level representing typical basal expression. Deviations of target protein abundances from this HK baseline were evaluated using a Z-test applied to the log10-transformed iBAQ intensities; proteins with *p* < 0.05 were considered significantly over- or under-represented relative to the housekeeping median.

### 2.10. Data

All data generated or analyzed during this study will be placed in open public databases during the review process. The raw sequencing data will be deposited in the NCBI Sequence Read Archive (SRA), and the complete genome assembly of the studied *Serratia rubidaea* 151 strain will be publicly available in the GenBank database since publication date as part of the BioProject ID PRJNA1426982. The mass spectrometry proteomics data will be deposited to the ProteomeXchange Consortium via the PRIDE partner repository.

## 3. Results

### 3.1. Species Identification

By species identification procedures, including time-of-flight mass spectrometry and standard morphological characterization, the clinical isolate was reliably identified as *Serratia rubidaea*; the extended results of these steps are mentioned in Supplementary File, Sections S1 and S2.

### 3.2. Antibiotics Resistance

The studied strain’s resistance properties were screened microbiologically against 27 antibiotics. The accounting of the results in accordance with the instructions of the kit manufacturer is reflected in expanded form in (Table 2). The phenotype of the studied strain is characterized by a high degree of resistance to antibiotics from various groups according to EUCAST and CLSI interpretation criteria.

**Figure 1:**
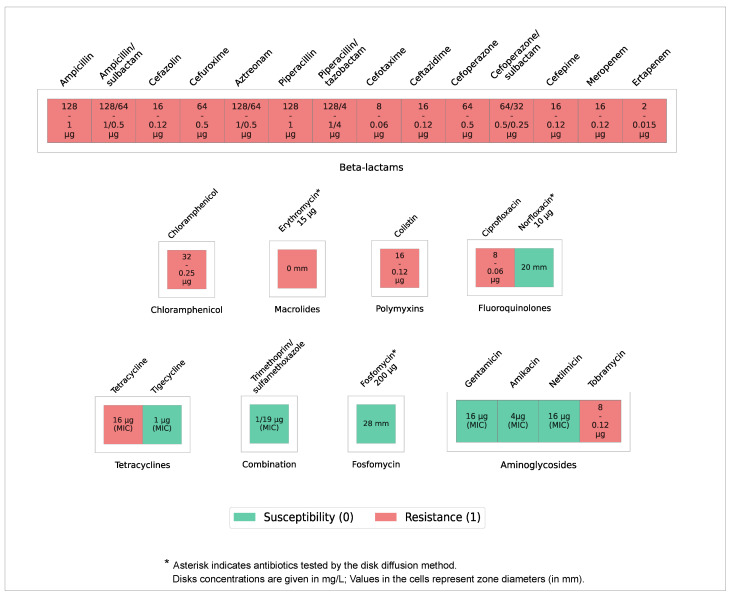
The studied strain antibiotic resistance examination results. Values in red cells reflect the maximum drug concentration used in the experiment (μg/mL; equivalent to mg/L) that failed to inhibit culture growth. Values in green cells reflect the MIC (μg/mL) that inhibits the growth of the test organism in the drug-containing medium. For antibiotics tested by the disk diffusion method, the amount of the drug per disk is mentioned in μg.

For the cephalosporin group, resistance to cefazolin, cefuroxime, cefotaxime, ceftazidime, cefoperazone, and cefepime was demonstrated; in the penicillin group, resistance was observed to ampicillin; in the aminoglycosides, resistance was found to tobramycin; in the carbapenems, resistance was detected to meropenem; in the monobactams, resistance was identified to aztreonam; in the polymyxins, resistance was noted to colistin; in the amphenicols, the strain is resistant to chloramphenicol; in the fluoroquinolones, resistance was observed to ciprofloxacin; and in the macrolides, resistance was found to erythromycin. Resistance to tetracycline was also detected, although the observed MIC values are borderline according to the interpretation criteria used in this study. The strain is also resistant to substances of the ureidopenicillin group, for example, piperacillin and others. Although a broad resistance profile to various antimicrobial drugs was revealed, the strain demonstrated sensitivity to gentamicin, netilmicin, and amikacin from the aminoglycoside group, and tigecycline, fosfomycin, and a mixture of trimethoprim and sulfamethoxazole, belonging to the diaminoprimidine/sulfonamide group, as well as to norfloxacin from the fluoroquinolone group (Figure 1).

Based on the results of the antibiotic resistance study, the usage of some cell wall protein (peptidoglycan) synthesis inhibitors, including penicillins, cephalosporins, monobactams, and carbapenems, will be insufficiently effective in suppressing the studied strain. However, the inhibitor of cytoplasmic membrane synthesis (fosfomycin) in the tested concentration was effective despite the discovery of a potential resistance determinant in the genome. Inhibitors of nucleic acid synthesis (fluoroquinolones) demonstrated mixed efficacy. Drugs that inhibit microbial protein synthesis (aminoglycosides, tetracyclines, and chloramphenicol) appear promising for practical use, despite the questionable efficacy of erythromycin, which operates through the same mechanism of action.

Despite varying susceptibility to antibiotics of common classes, no phenotypic evidence of heteroresistance to any of the tested antibiotics was observed in broth microdilution or disk diffusion assays. Under our testing conditions, the studied strain appeared phenotypically homogeneous by commonly accepted criteria [32,33].

### 3.3. Profiling of Disinfectant Tolerance

To determine the sensitivity of the studied microorganism to biocide agents widely used in medical institutions and industries, as well as in personal hygiene, the survival rate of the strain was studied when surfaces were treated with various disinfectants. All the tested biocide agents’ concentrations are mentioned in the Material and Methods section.

According to the obtained results, the studied strain of *Serratia rubidaea* demonstrates complete resistance to the treatment of the medium with such biocides as 1% H_2_O_2_ and 0.01% NaDCC, susceptibility to 2% and 3% H_2_O_2_, as well as the following substances: 0.3% ADBAC, 0.05% PHMG, tertiary amine in concentrations of 0.2% and below, 0.03% NaDCC, 0.05% glutaraldehyde, and ethanol in concentrations of 40%, 60%, and 70% (Table 3). Based on this data, we are able to conclude that treatment of the nutrient medium with the listed substances in the specified concentrations does not allow for a reduction in the number of CFU of the studied microorganism registered during the experiment to zero.

The studied microorganism shows full sensitivity only to two biocide agents: glutaraldehyde at concentrations of 0.25% and 0.5%, as well as to ADBAC at a concentration of 0.5%. These results revealed high survival of the studied strain under treatment with chlorine-containing disinfectants and alcohols, including the most popular for disinfection process C_2_H_5_OH concentration up to 70%. Concentrations of 3% and lower were ineffective when treated with commonly used H_2_O_2_.

### 3.4. Phylogeny

To verify the results of the whole-genome assembly, kmer prevalence analysis was performed, which mentioned *Serratia rubidaea* FDAARGOS_926 (NCBI RefSeq, GCF_016026735.1) as the closest reference organism, with a reference coverage of 72%. This result is consistent with the species identification of the culture obtained using MALDI-TOF MS.

The identification of the closest related strains using the generally known MLST multilocus typing scheme demonstrated a partial match of the target alleles with profiles available in the public PubMLST database. Based on this analysis, the most relevant MLST profile for our strain does not match any defined MLST type currently present in the database. Thus, close variants were found in the database for the *adk*, *fumC*, *gyrB*, *icd*, and *mdh* target genes. The closest related strain in terms of MLST profile is *Serratia rubidaea* XU1 (NCBI RefSeq, GCF_029087285.2), with the scheme *adk(87)*, *fumC(115)*, *gyrB(96)*, *icd(92)*, *mdh(96)*, and *recA(91)*. According to NCBI Refseq database, the XU1 strain was isolated in China in 2018 from soil samples.

However, the sequence of the *recA* gene of XU1 deposited in pubMLST is only 508 nucleotides long, and in BLAST (v2.16.0+) nucleotide alignment it maps with 99% identity onto a 508 nucleotide fragment of our strain’s *recA* gene, which has a total length of 1053 nucleotides. To further validate this observation, we performed an extended BLAST search of the *recA* sequence of our strain against the NCBI core_nt nucleotide database. This analysis revealed multiple matches to *Serratia rubidaea* strains with 98–99% identity. The closest match for the *recA* gene in the NCBI Nucleotide database is *Serratia rubidaea* SY163. According to NCBI GenBank database, the SY163 strain was isolated in Japan in 2020 from plant samples.

**Figure 2:**
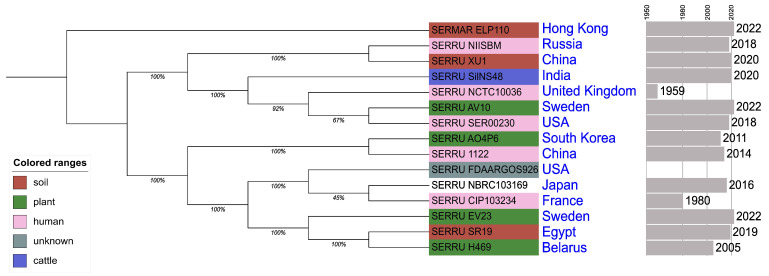
The phylogenetic tree built with pangenome analysis data. SERRU—*Serratia rubidaea*, SERMAR—*Serratia marcescens*. SERRU_NIISBM—studied strain, *Serratia rubidaea* 151. The color indicates the isolate source; the country and year of isolation of the reference sequences used in the analysis are indicated on the right; and the meanings below branches demonstrate that 45–100% branches support values.

The tree obtained with the pangenome analysis results presented in Figure 2. Metadata about sequences mentioned in Figure 2 can be found in the Material and Methods section. The obtained phylogenetic tree is consistent with the results of MLST-typing and species identification based on physical and morphological features. The studied strain, *Serratia rubidaea* 151, is a member of a clade formed by multiple representatives of the species *Serratia rubidaea*. This clade contains SERRU_NIISBM and the closest strain of *Serratia rubidaea* XU1, based on MLST typing. This indicates the similarity of the genomic structure of not only the genes included in the MLST typing scheme but also regions belonging to a broader set of genes. The outgroup is formed by a representative of the reference strain of the best-studied species of the genus *Serratia*—*Serratia marcescens* strain ELP1.10 (NCBI RefSeq, GCF_030291735.1).

### 3.5. Genome Structure

To identify genetic factors potentially responsible for epidemiologically significant properties such as antibiotic resistance and high-level disinfectants tolerance, we performed whole-genome sequencing of this strain.

Bacterial genome reads were generated using two sequencing technologies. The average read length obtained from ONT sequencing was 6.3 kbp, with a total of 2,206,571 reads and an average read quality by Phred Score around 20.55. The amount of reads with >Q20 Phred Score is 69.6%. The second-generation reads obtained using MGI sequencing were 150 bp, with a total of 4.6 million reads per sample and an average Phred Score of 30 to 35. The percentage of reads with a Phred Score > Q30 is 92.5%.

The genome assembly sequence included two circular closed replicons representing a circular chromosome (C1_chromosome; 5,076,905 bp) and a circular plasmid (C2_plasmid1; 79,106 bp). The total length of the genome sequence assembly is approximately 5.2 Mb. Assembly completeness is 98.8% by BUSCO results. Annotation revealed 4831 genes and 4772 coding sequences (CDS) in the assembly. Although 98.05% of the genes and 97.86% of the CDS are located on the chromosome, the plasmid also contained a cluster of *tra* genes required for the conjugation process, as well as *rep* genes (*repA*, *repB*, and *repC*), responsible for the synthesis of the corresponding replication proteins.

#### 3.5.1. Multiple Antibiotic Resistance Determinants

Genome analysis has allowed us to identify a number of determinants that potentially explain the antimicrobial resistance phenotype.

Several determinants in the genome of the studied strain represent a toxic resistance mechanism known as an efflux pump. The studied strain contains determinants belonging to efflux systems of various families. The first one is a chromosomal operon *sdeXY*, which encodes elements of the Resistance–Nodulation–Cell Division (RND-family) efflux pump SdeXY [34]. Another example of this family identified within the studied microorganism’s chromosome is *acrRAB* antimicrobial mechanisms of these types have affinity for a wide range of substrates and can provide multiple resistance properties, such as resistance to tigecycline, tetracycline, ciprofloxacin, and cefpirome, erythromycin, and norfloxacin, acriflavin, and others.

The other efflux family, ATP-binding cassette (ABC) antibiotic efflux pump, represented by *msbA* gene and *macAB* genes complex associated with macrolide, aminoglycoside, and polymyxine resistance [35,36], as well as *smdAB* efflux pumps, associated with fluoroquinolones and tetracycline resistance in *Serratia marcencens* [37].

There is also a chromosomal gene *mdtIJ* complex encoded small multidrug resistance (SMR) antibiotic efflux pump family transporter’s subunits and its silencing regulator gene *hns*, the encoded nucleoid-structuring protein H-NS [38]. Also, some of the major facilitator superfamily (MFS) antibiotic efflux pump examples were identified: the *emrR* region, also known as *mprA* encoded transcriptional repressor for the *emrRAB* operon where the *emrA* and *emrB* components lay downstream on the same chromosome [39,40], and the *marR* gene which is also a regulator for the *marRAB* [41] operon similar to *emrRAB*; however, *marAB* components weren’t identified within our study. Besides efflux pumps, there are several gene-encoding enzymes associated with resistance. An example is the *fos* gene, found on the chromosome, which encodes a protein that potentially provides increased resistance to fosfomycin (fosfomycin thiol transferase). The similarity of the amino acid sequence of the *fos* gene product with the sequence of the FosA protein from *Klebsiella pneumoniae* (SMTL ID: 5v91.1), calculated using the normalized BLOSUM62 substitution matrix [42,43], is 49% (seq_similarity). In addition, the use of InterproScan made it possible to identify in the structure of the *fos* gene product of the studied genome the glyoxalase domain (IPR004360), characteristic of the FosA and FosB proteins associated with the phenotype of resistance to fosfomycin in *Klebsiella pneumoniae* and *Escherichia coli* [44,45].

Another interesting cluster is the *arn*-cluster. The *arnAEFT* genes potentially mediate the synthesis activity of a protein similar to phosphoethanolamine transferase, which is involved in lipid A modification along with PhoP/PhoQ two-component systems and its MgrB regulator, which were all found in the *Serratia rubidaea* 151 genome. These determinants are associated with resistance to polymyxins, particularly colistin and polymyxin B, according to the CARD database and literature [6,43,45].

In addition to those mentioned, one more chromosomally encoded element potentially providing resistance to β-lactam antibiotics is the inducible β-lactamase gene *ampC* (Ambler class C β-lactamase, blaAmpC), which potentially provides protection to bacteria against penicillins and some cephalosporins [46].

#### 3.5.2. Fluoroquinolone Resistance Determinants

Additionally, the *gyrA* and *parC* genes were examined for the presence of specific single mutations associated with resistance to fluoroquinolone drugs in representatives of the Enterobacteriaceae family.

In the studied strain, no amino acid substitutions were found in the *gyrA* gene relative to the *E. coli* strain ATCC25922 recommended by the Clinical and Laboratory Standards Institute (CLSI) for use as a reference strain in antibiotic susceptibility testing. However, *Serratia rubidaea* 151 contains two substitutions in the *parC* gene associated with a resistance phenotype according to the literature [47,48,49,50]. Taking into account a number of literary data for the *gyrA* and *parC* genes, a wider set of positions associated in the literature with the resistance phenotype was studied [51,52]. The results of this screening for all organisms studied in the work are presented in Table 4.

The described amino acid composition at these positions is usual for all reference organisms used in this work as “Reference set” (Section 2).

#### 3.5.3. Disinfectants Resistance Determinants

Many of the various families of efflux pumps, including mechanisms described in the section above, are known to be involved in resistance to multiple biocide agents. Specifically, AcrAB-TolC is also known as a method of ADBAC, cetrimide, tetraphenylphosphonium, co-trimoxazole chlorhexidine, and didecyldimethylammonium chloride resistance [53]. Besides the wide set of efflux mechanisms studied, strains represent OmpA, OmpC, OmpF, OmpW, and OmpX porins, where OmpA- and OmpC-encoded genes are presented in several non-fragmented copies.

Although the loss of the *ompF* porin in combination with the *ampC* gene causes meropenem resistance in clinical isolates of Serratia [6], in our case, meropenem resistance was observed in phenotypic testing even in the presence of the *ompF* gene and its activity.

Other genetic determinants that potentially enhance resistance to environmental stress factors—high levels of ${\mathrm{Zn}}^{2+}$, ${\mathrm{Cd}}^{2+}$, and potentially ${\mathrm{Fe}}^{2+}$, as well as ${\mathrm{As}}^{3+}$—were also detected on the chromosome and plasmid of the studied strain. These genes include the *fieF* gene, which encodes a cation efflux pump [54], and the *arsABCDR* gene cluster, which encodes a system of ATP-dependent arsenic efflux transporters [55]. The two consecutive copies of the *yhcN* gene identified encode the protein product YhcN (peroxide/acid stress response protein YhcN, InterPro ID: IPR047775), which is involved in overcoming oxidative stress [56].

### 3.6. Identified Determinants Expression Assessment

**Figure 3:**
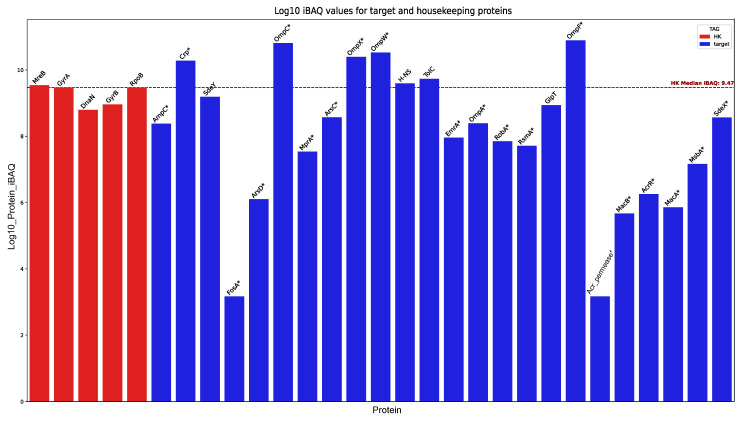
Comparison of iBAQ values for target and housekeeping (HK) proteins. All iBAQ values were log10-transformed for normalization. Statistical significance of the deviation from the HK mean baseline was assessed using a Z-test on the log10-transformed intensities; significant differences (*p* < 0.05) are indicated by an asterisk (*). Target proteins (blue) correspond to those encoded by genes associated with resistance- and tolerance-related features. Housekeeping proteins (red) comprise the set of reference proteins described in the Materials and Methods section.

In this study, we used proteomic analysis with the HPLC-MS/MS method to confirm the expression of several resistance determinants and their associated features. To evaluate the levels of detected proteins, the abundance of each target protein (iBAQ metric) was compared to the average baseline of stable housekeeping (HK) proteins. The statistical significance of the deviation from this baseline was determined using a Z-test on log10-transformed intensities, with significant differences (*p* < 0.05) marked by an asterisk (*). The results of this analysis can be found in Figure 3.

Proteomic profiling confirmed the basal constitutive expression of MFS and RND efflux pump components—specifically EmrA, SdeXY, MsbA, MacAB, and MprA—in the absence of antibiotics or initial environmental stressors. Expression evidence was also found for the permease subunit of the Acr cluster and its activator, RobA [57,58]. Furthermore, we detected the TolC membrane channel, which is essential for the functioning of toxic agent efflux systems. The expression of ArsCD components, part of the arsenic resistance cluster, was also validated.

Notably, the analysis confirmed the expression of all porins predicted from the genome: OmpA, OmpC, OmpF, OmpW, and OmpX. The detected abundance of OmpC, OmpF, OmpW, and OmpX was significantly higher (*p* < 0.05) than the average baseline of the HK group, whereas the abundance of OmpA* was significantly lower (*p* < 0.05) than this background threshold. With the exception of the aforementioned porins and the proteins SdeXY, GlpT, TolC, Crp, and H-NS—whose detected amounts approximated the baseline—all other proteins of interest were detected at levels significantly lower (*p* < 0.05) than the HK reference group.

Although the detected abundance for most proteins of interest was relatively low, it is necessary to consider the limitations of the detection method and the biological context of the cultivation conditions. The samples for proteomic analysis were collected in the absence of external stressors. Under such optimal conditions (nutrient-rich medium; no resource competition), the synthesis of proteins primarily dedicated to resistance mechanisms is likely downregulated in favor of systems driving active proliferation. Nevertheless, the reliable detection of unique peptides corresponding to these target proteins serves as robust evidence, confirming the physical presence and basal expression of the aforementioned resistance components in the studied samples.

**Figure 4:**
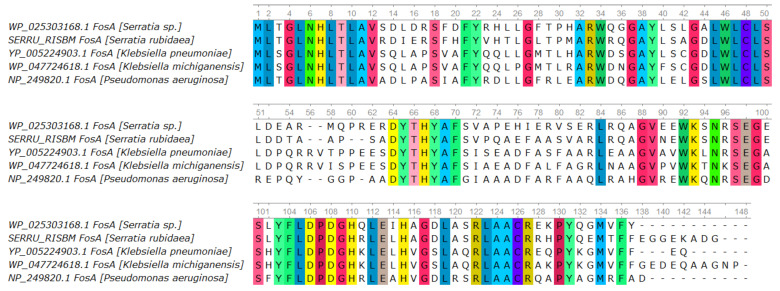
Comparison of the amino acid sequences of the FosA proteins from *S. rubidaea*, *S. marcescens*, *K. pneumoniae*, *K. michiganensis*, and *P. aeruginosa*. Amino acid alignment of the FosA protein sequences of the listed species. Amino acids are shown as standard one-letter IUPAC-IUBMB codes. Positions that are conserved across all species are color-coded.

Despite the detection in the studied genome of the chromosomal *fos* gene, encoding the FosA protein, known for its association with resistance to fosfomycin, the studied microorganism was sensitive to this drug according to the results of the testing by the disk diffusion method. Since proteomic analysis confirmed the expression of the FosA protein—the product of the *fos* gene in the studied strain—we compared its amino acid sequence with previously described FosA sequences from other bacteria [59]. Based on the obtained amino acid alignment results, a visualization was constructed (Figure 4), reflecting the preservation of the general structure of conserved domains in all of the listed organisms, including the studied strain. Given the absence of non-synonymous mutations in conserved sites in the FosA protein of the studied strain, the question regarding the potential limits of FosA activity or *fos* gene expression efficiency in the context of conferring resistance to various concentrations of fosfomycin for *Serratia rubidaea* remains open.

The proteomic data confirm the presence of the protein encoded by the *fos* gene in the bacterial cells of the studied strain. However, the detection of the protein alone is not sufficient to make definitive conclusions about its enzymatic activity or its contribution to resistance without directly assessing the response of the strain to fosfomycin exposure. According to the quantitative proteomic analysis (iBAQ), the level of the Fos protein under baseline cultivation conditions is lower than the median level for the proteins that we classified as housekeeping group (GyrAB, RpoB, MreB, and DnaN). This indirectly suggests a relatively low basal expression of this enzyme, but it may also be related to the absence of specific induction under the growth conditions used in our experiments.

## 4. Discussion

This study comprehensively characterized a clinical isolate of the opportunistic bacterium *Serratia rubidaea*. The strain 151 was isolated in a hospital environment and demonstrated resistance to a broad range of antibiotics and disinfectants from various groups. According to the criteria proposed in the paper [60] that standardized the use of the MDR, XDR, and PDR definitions, the investigated strain *S. rubidaea* (order Enterobacterales, genus Serratia) can be qualified as multidrug-resistant (MDR). Specifically, based on EUCAST interpretive criteria and results of our work, it is resistant (R) to more than one antimicrobial agent in more than three different antimicrobial categories. In the absence of information regarding the intrinsic resistance pattern specifically for *S. rubidaea*, we did not exclude any antimicrobial categories from our assessment other than those known to be intrinsically inactive against *Serratia marcescens* as the most studied member of the *Serratia* genus.

The resistance discovered for the *Serratia rubidaea* 151 shares many patterns with the determinants described for *Serratia marcescens*, which in turn is known to be an opportunist, posing a serious threat to neonatology and intensive care units. Despite the studied strain having mostly determinants related to the intrinsic resistance of the Serratia genus, the strain also contains a plasmid with a *tra*-gene cluster associated with the conjugative process, which indicates the potential of the strain as a reservoir for the accumulation of resistance features. However, the properties of *S. rubidaea* in terms of its ability to acquire resistance, as well as its adaptive tolerance range, remain to be studied. In addition, one of the methodological limitations of our study is the absence of internal quality control using certified reference strains. Such strains are not currently available to our laboratory within a reasonable regulatory and logistical framework. All susceptibility tests were nevertheless carried out in three replicates in strict accordance with EUCAST/CLSI recommendations and the manufacturers’ instructions, which partially mitigates this limitation.

The strain demonstrated complete resistance to all tested β-lactam antibiotics, including meropenem and ertapenem, which represent a subgroup of carbapenems—reserve drugs used when resistance to common β-lactam antibiotics is detected. The effectiveness of carbapenems is not repressed by classical β-lactamases, and, in particular, the AmpC cephalosporinase identified in the genome of the studied microorganism. Despite the complete resistance of the pathogen to the tested carbapenems (meropenem and ertapenem) in our work we did not find any known carbapenemase genes or their homologues in the *S. rubidaea* 151 genome.

When discussing resistance to β-lactam drugs, including carbapenems, it is essential to note a recent report of resistance to combinations of beta-lactam drugs with bacterial enzyme inhibitors, associated with the RND family of efflux mechanisms. We observed a similar effect in our study: the studied strain demonstrated complete resistance to a broad spectrum of β-lactam antibiotics, including carbapenems and combinations with β-lactamase inhibitors (e.g., tazobactam), in the context of the presence of various efflux mechanisms (*sdeXY* and *acrAB*) [61].

An interesting result of the resistance screening was the observation of partial resistance to some aminoglycoside and fluoroquinolone drugs. Among the aminoglycosides, tobramycin was ineffective in inhibiting culture growth, although gentamicin, amikacin, and netilmicin at different concentrations successfully inhibited the growth of *S. rubidaea*.

Aminoglycoside inhibition strategies include changes in the structure of the *16S rRNA* molecule—this mechanism is associated with bacterial modifying enzymes, such as *16S rRNA* methyltransferases (16S-RMTases) [62], and the transformation of the antibiotic molecule by aminoglycoside-modifying bacterial enzymes (AMMEs) of various classes. Along with this, background data on the manifestation of co-resistance within the aminoglycoside group vary worldwide. Cases of co-resistance to gentamicin and other aminoglycosides have been repeatedly recorded, including in samples obtained from clinical isolates [63]; however, on the other hand, aminoglycosides exhibit varying sensitivity to the enzymes that inactivate them [64]. The phenotype of susceptibility to gentamicin, amikacin, and netilmicin with resistance to tobramycin that we obtained is only partly consistent with literature data due to the same resistance level being usually observed for tobramycin and gentamicin in clinical isolates [65]. Also, we can conclude that the phenotype observed in our study does not coexist with aminoglycoside-resistance phenotypes among Russian clinical samples associated with aminoglycoside-modifying enzymes [66]. The reason may lie either in the variability of known aminoglycoside-modifying enzymes or in the activity of some homologues of known enzymes in the studied strain, which performs the same functions providing the phenotype of partial dose-dependent resistance to aminoglycosides. On the other hand, increased sensitivity to aminoglycosides has been associated in the literature with reduced expression of the post-translational factor RsmA, which is also associated with virulence factor production in *P. aeruginosa* [67,68]. The RsmA-encoding gene was found on the chromosome of *S. rubidaea* during the present study and RsmA protein also was detected in our analysis of proteomic data.

Of the two fluoroquinolones tested, the strain exhibited complete resistance to ciprofloxacin while being sensitive to norfloxacin. In the literature, particular attention is paid to serine (Ser) substitutions at position 80 (S80) and glutamine (Glu) substitutions at position 84 (E84) of the *parC* gene; additionally, asparagine (Asp) substitutions at position 87 (D87) and serine at position 83 (S83) in the *gyrA* gene are considered indicators of resistance to fluoroquinolones in bacteria. The studied strain *S. rubidaea* 151 contains both substitutions in the *parC* gene, but does not contain any substitution at position 83 of the *gyrA* gene. Despite this, the studied strain demonstrates a high level of resistance to ciprofloxacin at a concentration of 8 μg/mL at the same time as susceptibility to norfloxacin at a concentration of 10 μg/mL. Given the similarity of the chemical structures of the drugs and their molecular action, the resistance to ciprofloxacin that is demonstrated may be due to the use of a commercial panel at a concentration lower than that of norfloxacin, which was tested for susceptibility using the disk method. Thus, increasing the tested ciprofloxacin concentration up to 10 μg/mL could potentially lead to the susceptibility phenotype with both tested fluoroquinolones. On the one hand, whole-genome sequencing and proteomic analysis under baseline conditions demonstrated the presence of several efflux systems (including *sdeXY*, *acrRAB*, *macAB* and *emrAB*), which could contribute to decreased intracellular fluoroquinolone concentrations. On the other hand, we identified substitutions in *parC* in the absence of *gyrA* mutations, indicating a role for target modification. These observations suggest that both target modification and efflux are likely to contribute to the fluoroquinolone resistance phenotype; however, in the absence of efflux inhibition assays or targeted gene disruption experiments, we are unable to determine their relative quantitative impact.

A similar limitation concerning the elucidation of the contribution of each individual determinant to the strain’s phenotype was encountered during the analysis of the fosfomycin-related results. Since the overall structure of the *fos* gene in the studied strain includes conservative domains, and, according to the proteomic analysis, the protein FosA was detected in low abundance compared to the HK-group, we decided to analyze the proteins of the fosfomycin uptake system (UhpT and GlpT proteins) to further address observed fosfomycin susceptibility phenotype. In the genome of the studied strain, no *uhpT* gene or close homologues were identified, whereas the *glpT* gene, encoding the glycerol-3-phosphate transporter, is present in an intact form. In light of published data on the roles of UhpT and GlpT in fosfomycin uptake by Gram-negative bacteria [69,70], we hypothesize that the observed phenotype—the presence of the *fos* gene, the absence of *uhpT* or its close homologue, and an intact *glpT* gene encoding a GlpT protein detected in our proteomic data—may reflect a combination of the limited efficiency of the enzyme encoded by *fos*, and the secondary role of UhpT-mediated transport compared with the predominant role of GlpT in active fosfomycin uptake in this strain. The precise balance between these factors in our isolate can only be determined through dedicated functional experiments, which are beyond the scope of the present study.

Some determinants in the studied genome include the complete *macAB* efflux operon-encoding genes functioning in a complex with TolC, as well as other RND-family efflux mechanisms associated in the literature with resistance to macrolide antibiotics, in particular erythromycin [2], which is consistent with the phenotype of *S. rubidaea* observed in our research.

In some studies, the joint work of the EmrR+OmpW pump is also associated with resistance to tetracyclines [71], but it is not confirmed by resistance phenotype in the present study.

In the present study, a phenotype was obtained that is only partially consistent with the data on the antibiotic activity of the SdeXY transporter system, since bacteria expressing the SdeXY-HasF system showed resistance to tigecycline, cefpirome (a fourth-generation cephalosporin, a β-lactam antibiotic), and ciprofloxacin [72]. In our study encoding HasF gene was not identified, and the observed phenotype included resistance to ciprofloxacin and cefepime (a drug of the same group and generation as cefpirome) with sensitivity to tigecycline. These results may be explained by the insufficient efficiency of the antitoxic function of the SdeXY system in the absence of the HasF component, or by the achievement of resistance to these antibiotics due to the work of other determinants found in the genome, different from the SdeXY-HasF efflux pump.

The current study highlights a concerning finding: the broad resistance profile observed in a common contaminant and opportunistic pathogen, *S. rubidaea*, appears to be largely attributable to the activity of multiple efflux pumps. Although efflux-mediated resistance is particularly challenging to combat—as a single pump can extrude a wide range of antimicrobial agents and disinfectants, resulting in significant cross-resistance—these pumps are often energetically costly for the bacterial cell due to their ATP-dependent mechanism. Therefore, while their exact evolutionary fitness cost requires further investigation, their acquisition provides a clear survival advantage in the selective environment of a healthcare facility.

## 5. Conclusions

Beyond the broad intrinsic resistance in studied isolate, the high epidemiological significance of *Serratia* spp. lies in their capacity for persistence on medical equipment and biofilm formation ability [8,10]. The combination of multiple resistance to antibiotics and disinfectants with biofilm formation ability could initiate resilient microbial niches in hostile environments. Within such niches, multi-resistance bacterium could facilitate the persistent survival of a heterogeneous microbiota. Moreover, tight location of diverse species under sublethal stress could promote the selection of the tolerance phenotype and the horizontal transfer processes. In the end, environments where such resistant strains establish permanently could evolve into the reservoirs for the accumulation of multidrug resistance and associated pathogenic flora. Thus, monitoring these organisms is critical, as their presence signals a need for stricter antimicrobial control and disinfectant prevention in healthcare settings—even against microbiota lacking obvious classical virulence factors.

## Figures and Tables

**Table 1:** Strains used in the comparative genomic analysis.

ID	NCBI RefSeq Accession	Organism	Strain	Location	Collection Date	Isolation Source
SERMAR_ELP110	GCF_030291735.1	*Serratia marcescens*	ELP1.10	Hong Kong	2022	soil
SERRU_BCZJ01	GCF_001598675.1	*Serratia rubidaea*	NBRC_103169	Japan, Osaka	2016	unknown
SERRU_H469	GCF_021498225.1	*Serratia rubidaea*	H469	Belarus, Gdansk	2005	plant
SERRU_NCTC10036	GCF_900638005.1	*Serratia rubidaea*	NCTC10036	United Kingdom	1959	human skin
SERRU_FDA_926	GCF_016026735.1	*Serratia rubidaea*	FDAARGOS_926	USA	unknown	unknown
SERRU_XU1	GCF_029087285.2	*Serratia rubidaea*	XU1	China, Xinjiang	2020	soil
SERRU_1122	GCF_001572725.1	*Serratia rubidaea*	1122	China, Beijing	2014	human sputum
SERRU_AO4P6	GCF_027557545.1	*Serratia rubidaea*	AO4-P6	South Korea	2011	plant
SERRU_SR19	GCF_042847125.1	*Serratia rubidaea*	SR19	Egypt, Gahrbia	2019	soil
SERRU_CIP103234	GCF_001304675.1	*Serratia rubidaea*	CIP 103234	France, Paris	1980	human
SERRU_EV23	GCF_028462665.1	*Serratia rubidaea*	EV23	Sweden	2022	plant
SERRU_AV10	GCF_028462745.1	*Serratia rubidaea*	AV10	Sweden	2022	plant
SERRU_SER00230	GCF_015999165.1	*Serratia rubidaea*	SER00230	USA, Pennsylvania	2018	human sputum
SERRU_SilNS48	GCF_035792815.1	*Serratia rubidaea*	Sil NS 48	India	2020	cattle

**Table 2:** Antimicrobial susceptibility testing results for the studied *Serratia rubidaea* strain. S: susceptible, R: resistant, I: intermediate, and NS: non-susceptible. Concentrations are given in mg/L for MIC-based breakpoints and in μg for disk contents.

Antimicrobial	Agent	MIC Criteria (mg/L)			Disk Diffusion (µg/mm)			Result	Interpretation Source
		EUCAST S≤	EUCAST R≥	Our Value	EUCAST Disk/Zone	Our Disk/Zone		(S/I/R)	
Aminoglycosides	Gentamicin	2	2	16	–	–		R	EUCAST 2025
	Tobramycin	2	2	8	–	–		R	EUCAST 2025
	Amikacin	8	8	4 (MIC)	–			S	EUCAST 2025
	Netilmicin	2	8	16	IE	IE		R	Manufacturer instruction, EUCAST 2018
Carbapenems	Ertapenem	0.5	0.5	0.015 (MIC)	–	–		S	EUCAST 2025
	Meropenem	2	8	16	–	–		R	EUCAST 2025
Non-extended spectrum cephalosporins (1st–2nd gen.)	Cefazolin	0.001	4	16	–	–		R	EUCAST 2025
	Cefuroxime	8	8	64	–	–		R	EUCAST 2025
Extended spectrum cephalosporins (3rd–4th gen.)	Cefotaxime /Ceftriaxone	1	2	8	–	–		R	EUCAST 2025
	Ceftazidime	1	4	16	–	–		R	EUCAST 2025
	Cefepime	1	4	16	–	–		R	EUCAST 2025
	Cefoperazone	16	64	64	–	–		R	Manufacturer instruction, CLSI 2018
	Cefoperazone /Sulbactam	–	–	64/32	–	–		–	No criteria
Fluoroquinolones	Ciprofloxacin	0.25	0.5	8	–	–		R	EUCAST 2025
	Norfloxacin	–	–	–	10/24	10/20		R	EUCAST 2025
Folate pathway inhibitors	Trimethoprim /sulfamethoxazole	2/38	4/76	1/19 (MIC)	–	–		S	EUCAST 2025
Glycylcyclines	Tigecycline	1	4	1	–	–	–	S	Manufacturer instruction, EUCAST 2020
Monobactams	Aztreonam	1	4	16	–	–	–	R	EUCAST 2025
Penicillins	Ampicillin	8	8	128	–	–	–	R	EUCAST 2025
	Ampicillin /sulbactam	8/4	8/4	128/64	–	–	–	R	EUCAST 2025
	Piperacillin	8	8	128	–	–	–	R	EUCAST 2025
	Piperacillin /tazobactam	8/4	8/4	128/4	–	–	–	R	EUCAST 2025
Phenicols	Chloramphenicol	8	16	32	–	–	–	R	Manufacturer instruction, EUCAST 2020
Phosphonic acids	Fosfomycin	8	8	–	200/24	200/28	–	S	EUCAST 2025
Polymyxins	Colistin	2	2	16	–	–	–	R	EUCAST 2025
Tetracyclines	Tetracycline	4	16	16	–	–	–	R	Manufacturer instruction, CLSI 2020
Macrolides	Erythromycin	–	–	–	–	15/0	–	R	No criteria

**Table 3:** *Serratia rubidaea* strain’s disinfectants tolerance study results.

Disinfectant	Petri Dishes						Culture Tubes					
	%	CFU	%	CFU	%	CFU	%	CFU/mL	%	CFU/mL	%	CFU/mL
H_2_O_2_	1.00	1000>	2.00	100>	3.00	4	1.00	$9\times 10^{8}$	2.00	$6\times 10^{8}$	3.00	$6\times 10^{8}$
ADBAC	0.1	5	0.3	3	0.5	0	0.1	$6\times 10^{8}$	0.3	$6\times 10^{8}$	0.5	0
PHMG	0.01	20	0.02	7	0.05	2	0.01	$6\times 10^{8}$	0.02	$6\times 10^{8}$	0.05	$6\times 10^{8}$
Triam.	0.05	12	0.1	4	0.2	1	0.05	$6\times 10^{8}$	0.1	$6\times 10^{8}$	0.2	$6\times 10^{8}$
NaDCC	0.01	1000>	0.03	4	0.05	0	0.01	$6\times 10^{8}$	0.03	$6\times 10^{8}$	0.05	$1\times 10^{8}$
Glutaraldehyde	0.05	1	0.25	0	0.5	0	0.05	$6\times 10^{8}$	0.25	0	0.5	0
C_2_H_5_OH	40	2	60	11	70	8	40	$6\times 10^{8}$	60	$6\times 10^{8}$	70	$6\times 10^{8}$

**Table 4:** Investigation of QRDR region substitutions associated with the resistance phenotype. Strains names correspond the ID values of the “Reference set” mentioned in the Material and Methods section.

	gyrA			parC									
Strain	81	83	87	13	16	33	37	50	59	60	80	84	133
*E. coli* ATCC 25922	G (Gly)	S (Ser)	D (Asp)	A (Ala)	E (Glu)	R (Arg)	F (Phe)	V (Val)	N (Asn)	A (Ala)	P (Pro)	S (Ser)	S (Ser)
*SERMAR_ELP110*	G (Gly)	S (Ser)	D (Asp)	P (Pro)	T (Thr)	R (Arg)	Y (Tyr)	I (Ile)	T (Thr)	N (Asn)	P (Pro)	S (Ser)	A (Ala)
*SERRU_FDAARGOS926*	G (Gly)	S (Ser)	D (Asp)	P (Pro)	T (Thr)	R (Arg)	Y (Tyr)	I (Ile)	S (Ser)	N (Asn)	P (Pro)	S (Ser)	A (Ala)
*SERRU_XU1*	G (Gly)	S (Ser)	D (Asp)	P (Pro)	T (Thr)	R (Arg)	Y (Tyr)	I (Ile)	S (Ser)	N (Asn)	P (Pro)	S (Ser)	A (Ala)
*Serratia rubidaea* 151	G (Gly)	S (Ser)	D (Asp)	P (Pro)	T (Thr)	R (Arg)	Y (Tyr)	I (Ile)	S (Ser)	N (Asn)	P (Pro)	S (Ser)	A (Ala)

## Data Availability

The original data presented in the study are openly available in NCBI Sequence Read Archive (SRA) under BioProject accession number PRJNA1426982. The complete genome assembly of the studied *Serratia rubidaea* 151 strain is available upon request.

## Supplementary Materials

The following supporting information can be downloaded at: https://www.mdpi.com/article/10.3390/microorganisms14050988/s1, Supplementary Materials Section S1: Species identification; Section S2: Morphological properties; Section S3: Antibiotics resistance; Figure S1: Morphological assessment using the MS-7-ZOOM stereoscopic trinocular microscope. Microscope magnification: 0.67–4.5x; Figure S2: Morphological assessment using the MS-7-ZOOM stereoscopic trinocular microscope. Microscope magnification: 0.67–4.5x; Table S1: Disk diffusion method results. Disk content represented in µg per disk; Table S2: Antimicrobial susceptibility tested panels for the studied strain (Part 1). Concentrations are mentioned in µg/mL; Table S3: Antimicrobial susceptibility tested panels for the studied strain (Part 2). Concentrations are mentioned in µg/mL.

## References

[1] Karaman R., Jubeh B., Breijyeh Z. (2020). Resistance of Gram-Positive Bacteria to Current Antibacterial Agents and Overcoming Approaches. Molecules.

[2] Li X.Z., Plésiat P., Nikaido H. (2015). The challenge of efflux-mediated antibiotic resistance in Gram-negative bacteria. Clin. Microbiol. Rev..

[3] Zhuang M., Achmon Y., Cao Y., Liang X., Chen L., Wang H., Siame B.A., Leung K.Y. (2021). Distribution of antibiotic resistance genes in the environment. Environ. Pollut..

[4] Chinemerem Nwobodo D., Ugwu M.C., Oliseloke Anie C., Al-Ouqaili M.T.S., Chinedu Ikem J., Victor Chigozie U., Saki M. (2022). Antibiotic resistance: The challenges and some emerging strategies for tackling a global menace. J. Clin. Lab. Anal..

[5] Jonas O.B., Irwin A. (2017). Drug-Resistant Infections: A Threat to Our Economic Future.

[6] Tavares-Carreon F., De Anda-Mora K., Rojas-Barrera I.C., Andrade A. (2023). Serratia marcescens antibiotic resistance mechanisms of an opportunistic pathogen: A literature review. PeerJ.

[7] Guel-Gomez M., Angulo-Zamudio U.A., Leon-Sicairos N., Flores-Villaseñor H., Mendívil-Zavala E., Plata-Guzmán A., Martinez-Garcia J.J., Angulo-Rocha J., Ochoa-Espinoza R., Crespo-Palazuelos P. (2023). Outbreak of Serratia marcescens in the neonatal intensive care unit of a tertiary care hospital in Mexico. Adv. Med..

[8] Mehdi A., Trifi A., Abbes S., Seghir E., Tlili B., Masseoud L., Noussair A., Ouhibi A., Battikh H., Zribi M. (2023). Bacteremia due to Serratia rubidaea in intensive care unit: A case series. J. Med. Case Rep..

[9] Taxt A.M., Eldholm V., Kols N.I., Haugan M.S., Raffelsberger N., Asfeldt A.M., Ingebretsen A., Blomfeldt A., Kilhus K.S., Lindemann P.C. (2025). A national outbreak of Serratia marcescens complex: Investigation reveals genomic population structure but no source, Norway, June 2021 to February 2023. Eurosurveillance.

[10] Ursua P.R., Unzaga M.J., Melero P., Iturburu I., Ezpeleta C., Cisterna R. (1996). Serratia rubidaea as an invasive pathogen. J. Clin. Microbiol..

[11] Federal Service for Surveillance in the Sphere of Consumer Rights Protection and Human Well-Being (Rospotrebnadzor) (2024). MR 3.1.0346-24: Organization and Conduct of Microbiological Monitoring in Medical Organizations Methodological Recommendations.

[12] Semina N.A., Sidorenko S.V., Rezvan S.P., Grudinina S.A., Strachunsky L.S., Stetsyuk O.U., Kozlov R.S., Eidelynteyn M.V., Vedmina E.A., Stolyarova L.G. (2004). Antimicrobial Susceptibility Testing Guidelines.

[13] Igonina E.P., Ilyina E.N., Fedorova L.S., Kovalchuk S.N., Arkhipova A.L., Demina Y.V., Eremeeva N.I., Ilyakova A.V., Serov A.A. (2024). Assessing the Susceptibility of Microorganisms Circulating in Healthcare Facilities to Disinfectants.

[14] Rutala W.A., Weber D.J. (2024). Guideline for Disinfection and Sterilization in Healthcare Facilities.

[15] Andrews S. FastQC: A Quality Control Tool for High Throughput Sequence Data, 2010. http://www.bioinformatics.babraham.ac.uk/projects/fastqc/.

[16] De Coster W., Rademakers R. (2023). NanoPack2: Population-scale evaluation of long-read sequencing data. Bioinformatics.

[17] Wick R.R., Judd L.M., Gorrie C.L., Holt K.E. (2017). Unicycler: Resolving bacterial genome assemblies from short and long sequencing reads. PLoS Comput. Biol..

[18] Bouras G., Grigson S.R., Papudeshi B., Mallawaarachchi V., Roach M.J. (2024). Dnaapler: A tool to reorient circular microbial genomes. J. Open Source Softw..

[19] Manni M., Berkeley M.R., Seppey M., Simão F.A., Zdobnov E.M. (2021). BUSCO Update: Novel and Streamlined Workflows along with Broader and Deeper Phylogenetic Coverage for Scoring of Eukaryotic, Prokaryotic, and Viral Genomes. Mol. Biol. Evol..

[20] Seemann T. (2014). Prokka: Rapid prokaryotic genome annotation. Bioinformatics.

[21] Li W., O’Neill K.R., Haft D.H., DiCuccio M., Chetvernin V., Badretdin A., Coulouris G., Chitsaz F., Derbyshire M.K., Durkin A.S. (2021). RefSeq: Expanding the Prokaryotic Genome Annotation Pipeline reach with protein family model curation. Nucleic Acids Res..

[22] Tatusova T., DiCuccio M., Badretdin A., Chetvernin V., Nawrocki E., Zaslavsky L., Lomsadze A., Pruitt K., Borodovsky K., Ostell J. (2016). NCBI prokaryotic genome annotation pipeline. Nucleic Acids Res..

[23] Feldgarden M., Brover V., Gonzalez-Escalona N., Frye G.R., Haendiges J., Haft D.H., Hoffmann D., Pettengill P.B., Prasad A.B., Tillman G.E. (2021). AMRFinderPlus and the Reference Gene Catalog facilitate examination of the genomic links among antimicrobial resistance, stress response, and virulence. Sci. Rep..

[24] Alcock B.P., Huynh W., Chalil R., Smith K., Raphenya A.R., Wlodarski M.A., Edalatmand A., Petkau A., Syed A., Tsang K. (2023). CARD 2023: Expanded curation, support for machine learning, and resistome prediction at the Comprehensive Antibiotic Resistance Database. Nucleic Acids Res..

[25] Larsen M.V., Cosentino S., Lukjancenko O., Saputra D., Rasmussen S., Hasman H., Sicheritz-Pontén T., Aarestrup F.M., Ussery D.W., Lund O. (2014). Benchmarking of methods for genomic taxonomy. J. Clin. Microbiol..

[26] Seemann T. (2023). mlst: Multi-Locus Sequence Typing. GitHub Repository. https://github.com/tseemann/mlst.

[27] Jolley K.A., Bray J.E., Maiden M.C.J. (2018). Open-access bacterial population genomics: BIGSdb software, the PubMLST.org website and their applications. Wellcome Open Res..

[28] Tonkin-Hill G., MacAlasdair N., Ruis C., Weimann A., Horesh G., Lees J.A., Gladstone R.A., Lo S., Beaudoin C., Floto R.A. (2020). Producing polished prokaryotic pangenomes with the Panaroo pipeline. Genome Biol..

[29] Minh B.Q., Schmidt H.A., Chernomor O., Schrempf D., Woodhams M.D., von Haeseler A., Lanfear R. (2020). IQ-TREE 2: New Models and Efficient Methods for Phylogenetic Inference in the Genomic Era. Mol. Biol. Evol..

[30] Letunic I., Bork P. (2024). Interactive Tree Of Life (iTOL) v6: Recent updates to the phylogenetic tree display and annotation tool. Nucleic Acids Res..

[31] Teo G.C., Polasky D.A., Yu F., Nesvizhskii A.I. (2020). A fast deisotoping algorithm and its implementation in the MSFragger search engine. J. Proteome Res..

[32] Bardet L., Baron S., Leangapichart T., Okdah L., Diene S.M., Rolain J.M. (2017). Deciphering heteroresistance to colistin in a Klebsiella pneumoniae isolate from Marseille, France. Antimicrob. Agents Chemother..

[33] Landman D., Salamera J., Quale J. (2013). Irreproducible and uninterpretable polymyxin B MICs for Enterobacter cloacae and Enterobacter aerogenes. J. Clin. Microbiol..

[34] Chen J., Kuroda T., Huda M.N., Mizushima T., Tsuchiya T. (2003). An RND-type multidrug efflux pump SdeXY from *Serratia marcescens*. J. Antimicrob. Chemother..

[35] Shirshikova T.V., Sierra-Bakhshi C.G., Kamaletdinova L.K., Matrosova L.E., Khabipova N.N., Evtugyn V.G., Khilyas I.V., Danilova I.V., Mardanova A.M., Sharipova M.R. (2021). The ABC-Type Efflux Pump MacAB Is Involved in Protection of *Serratia marcescens* against Aminoglycoside Antibiotics, Polymyxins, and Oxidative Stress. mSphere.

[36] Greene N.P., Kaplan E., Crow A., Koronakis V. (2018). Antibiotic Resistance Mediated by the MacB ABC Transporter Family: A Structural and Functional Perspective. Front. Microbiol..

[37] Matsuo T., Chen J., Minato Y., Ogawa W., Mizushima T., Kuroda T., Tsuchiya T. (2008). SmdAB, a Heterodimeric ABC-Type Multidrug Efflux Pump in *Serratia marcescens*. J. Bacteriol..

[38] Leuzzi A., Di Martino M.L., Campilongo R., Falconi M., Barbagallo M., Marcocci L., Pietrangeli P., Casalino M., Grossi M., Micheli G. (2015). Multifactor Regulation of the MdtJI Polyamine Transporter in *Shigella*. PLoS ONE.

[39] Lomovskaya O., Lewis K., Matin A. (1995). EmrR is a negative regulator of the *Escherichia coli* multidrug resistance pump EmrAB. J. Bacteriol..

[40] Xiong A., Gottman A., Park C., Baetens M., Pandza S., Matin A. (2000). The EmrR protein represses the *Escherichia coli* emrRAB multidrug resistance operon by directly binding to its promoter region. Antimicrob. Agents Chemother..

[41] Seoane A.S., Levy S.B. (1995). Characterization of MarR, the repressor of the multiple antibiotic resistance (mar) operon in *Escherichia coli*. J. Bacteriol..

[42] Trivedi R., Nagarajaram H.A. (2019). Amino acid substitution scoring matrices specific to intrinsically disordered regions in proteins. Sci. Rep..

[43] Fernández L., Alvarez-Ortega C., Wiegand I., Olivares J., Kocíncová D., Lam J.S., Martínez J.L. (2013). Characterization of the polymyxin B resistome of *Pseudomonas aeruginosa*. Antimicrob. Agents Chemother..

[44] Klontz E.H., Tomich A.D., Günther S., Lemkul J.A., Deredge D., Silverstein Z., Shaw J.F., McElheny C., Doi Y., Wintrode P.L. (2017). Structure and Dynamics of FosA-Mediated Fosfomycin Resistance in *Klebsiella pneumoniae* and *Escherichia coli*. Antimicrob. Agents Chemother..

[45] Schumann A., Gaballa A., Yang H., Yu D., Ernst R.K., Wiedmann M. (2024). Site-selective modifications by lipid A phosphoethanolamine transferases linked to colistin resistance and bacterial fitness. mSphere.

[46] Sawa T., Kooguchi K., Moriyama K. (2020). Molecular diversity of extended-spectrum *β*-lactamases and carbapenemases, and antimicrobial resistance. J. Intensive Care.

[47] Dutta S., Kawamura Y., Ezaki T., Nair G.B., Iida K., Yoshida S. (2005). Alteration in the GyrA subunit of DNA gyrase and the ParC subunit of topoisomerase IV in Quinolone-resistant *Shigella dysenteriae* serotype 1 clinical isolates from Kolkata, India. Antimicrob. Agents Chemother..

[48] Vila J., Ruiz J., Marco F., Barcelo A., Goñi P., Giralt E., Anta T. (1994). Association between double mutation in gyrA gene of ciprofloxacin-resistant clinical isolates of *Escherichia coli* and MICs. Antimicrob. Agents Chemother..

[49] Vila J., Ruiz J., Goñi P., De Anta M.T. (1996). Detection of mutations in parC in quinolone-resistant clinical isolates of *Escherichia coli*. Antimicrob. Agents Chemother..

[50] Weigel L.M., Steward C.D., Tenover F.C. (1998). gyrA mutations associated with fluoroquinolone resistance in eight species of Enterobacteriaceae. Antimicrob. Agents Chemother..

[51] Moon D.C., Seol S.Y., Gurung M., Jin J., Choi C.H., Kim J., Lee Y., Cho D., Lee J. (2010). Emergence of a new mutation and its accumulation in the topoisomerase IV gene confers high levels of resistance to fluoroquinolones in *Escherichia coli* isolates. Int. J. Antimicrob. Agents.

[52] Dehbanipour R., Khanahmad H., Sedighi M., Bialvaei A.Z., Faghri J. (2019). High prevalence of fluoroquinolone-resistant *Escherichia coli* strains isolated from urine clinical samples. J. Prev. Med. Hyg..

[53] Kovalchuk S.N., Fedorova L.S., Ilina E.N. (2023). Molecular mechanisms of microbial resistance to disinfectants. Antibiot. Chemother..

[54] Grass G., Otto M., Fricke B., Haney C.J., Rensing C., Nies D.H., Munkelt D. (2005). FieF (YiiP) from *Escherichia coli* mediates decreased cellular accumulation of iron and relieves iron stress. Arch. Microbiol..

[55] Carlin A., Shi W., Dey S., Rosen B.P. (1995). The ars operon of *Escherichia coli* confers arsenical and antimonial resistance. J. Bacteriol..

[56] Lee J., Hiibel S.R., Reardon K.F., Wood T.K. (2010). Identification of stress-related proteins in *Escherichia coli* using the pollutant cis-dichloroethylene. J. Appl. Microbiol..

[57] Nakajima H., Kobayashi K., Kobayashi M., Asako H., Aono R. (1995). Overexpression of the robA gene increases organic solvent tolerance and multiple antibiotic and heavy metal ion resistance in *Escherichia coli*. Appl. Environ. Microbiol..

[58] White D.G., Goldman J.D., Demple B., Levy S.B. (1997). Role of the acrAB locus in organic solvent tolerance mediated by expression of marA, soxS, or robA in *Escherichia coli*. J. Bacteriol..

[59] Ito R., Mustapha M.M., Tomich A.D., Callaghan C., McElheny C.L., Mettus R.T., Shanks R., Sluis-Cremer N., Doi Y. (2017). Widespread Fosfomycin Resistance in Gram-Negative Bacteria Attributable to the Chromosomal fosA Gene. mBio.

[60] Magiorakos A.P., Srinivasan A., Carey R., Carmeli Y., Falagas M., Giske C., Harbarth S., Hindler J., Kahlmeter G., Olsson-Liljequist B. (2012). Multidrug-resistant, extensively drug-resistant and pandrug-resistant bacteria: An international expert proposal for interim standard definitions for acquired resistance. Clin. Microbiol. Infect..

[61] Chiang A.D., Dekker J.P. (2024). Efflux pump-mediated resistance to new beta lactam antibiotics in multidrug-resistant gram-negative bacteria. Commun. Med..

[62] Doi Y., Wachino J.I., Arakawa Y. (2016). Aminoglycoside Resistance: The Emergence of Acquired 16S Ribosomal RNA Methyltransferases. Infect. Dis. Clin. N. Am..

[63] Moellering R.C., Wennersten C., Kunz L.J., Poitras J.W. (1977). Resistance to gentamicin, tobramycin and amikacin among clinical isolates of bacteria. Am. J. Med..

[64] McManus M.C. (1997). Mechanisms of bacterial resistance to antimicrobial agents. Am. J. Health-Syst. Pharm..

[65] Gad G.F., Mohamed H.A., Ashour H.M. (2011). Aminoglycoside resistance rates, phenotypes, and mechanisms of Gram-negative bacteria from infected patients in upper Egypt. PLoS ONE.

[66] Reshedko G.K. (2001). Mechanisms of aminoglycoside resistance in nosocomial Gram-negative bacteria in Russia: Results of a multicenter study. Clin. Microbiol. Antimicrob. Chemother..

[67] Pessi G., Williams F., Hindle Z., Heurlier K., Holden M.T.G., Cámara M., Haas D., Williams P. (2001). The global posttranscriptional regulator RsmA modulates production of virulence determinants and N-acylhomoserine lactones in *Pseudomonas aeruginosa*. J. Bacteriol..

[68] Mulcahy H., O’Callaghan J., O’Grady E.P., Adams C., O’Gara F. (2006). The posttranscriptional regulator RsmA plays a role in the interaction between *Pseudomonas aeruginosa* and human airway epithelial cells by positively regulating the type III secretion system. Infect. Immun..

[69] Pavlova A.S., Bocharova Y.A., Kuleshov K.V., Podkolzin A.T., Chebotar I.V. (2021). Molecular determinants of antibiotic resistance in Salmonella enterica antibiotic resistance. J. Microbiol. Epidemiol. Immunobiol..

[70] Koulenti D., Timsit J.F. (2026). Fosfomycin Use in Treating Severe Difficult-to-Treat Gram-Negative Infections—A Comprehensive Review. Antibiotics.

[71] Beketskaia M.S., Bay D.C., Turner R.J. (2014). Outer Membrane Protein OmpW Participates with Small Multidrug Resistance Protein Member EmrE in Quaternary Cationic Compound Efflux. J. Bacteriol..

[72] Hornsey M., Ellington M.J., Doumith M., Hudson S., Livermore D.M., Woodford N. (2010). Tigecycline resistance in *Serratia marcescens* associated with up-regulation of the SdeXY-HasF efflux system also active against ciprofloxacin and cefpirome. J. Antimicrob. Chemother..

