# Supplementary material for: Characteristics of Serratia rubidaea Clinical Strain Revealed Multiple Resistance to Antibiotics and Disinfectants
Source: Microorganisms. 2026 Apr 28;14(5):988. doi: 10.3390/microorganisms14050988 (PMC13210221; doi:10.3390/microorganisms14050988)
Supplement: Supplementary file 1 [file microorganisms-14-00988-s001.zip › microorganisms-4194924-supplementary.pdf]

# Supplementary Materials:

## 1. Supplementary file S1: Extended RESULTS

*Section S1. Species identi ication*

The mass spectral profile obtained using MALDI-TOF MS revealed distinct peaks at m/z 7836.19, 4295.63, and 5364.82, which reliably correspond (scores 1.74–2.32) to the marker values of the reference species according to the associated software DL Mass Software, V1.1.1.0. Multiple matches with the spectra of reference organisms in the database confirm the correctness of the species identification.

*Section S2. Morphological properties*

To further identify and characterize the microorganism detected in the clinical isolate, a time-of-flight mass spectrometer was used, along with Gram staining and morphological characterization of the strain's colonies. Gram staining revealed Gram-negative, single, small, oval-shaped rods. Single colonies assessment after 24 hours of cultivation at 37±1°C in Endo differential-selective medium confirmed the presence of uniform dark pink, mucous, S-shaped colonies, surrounded by a clear visible halo of a darker base (Figure S1 and S2).

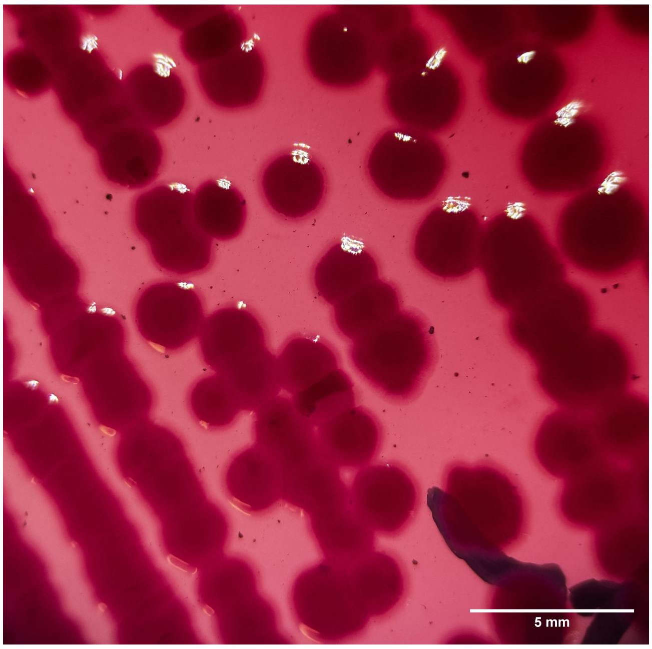


**Figure S1.** Morphological assessment using the MS-7-ZOOM stereoscopic trinocular microscope. Microscope magnification: 0.67-4.5x.

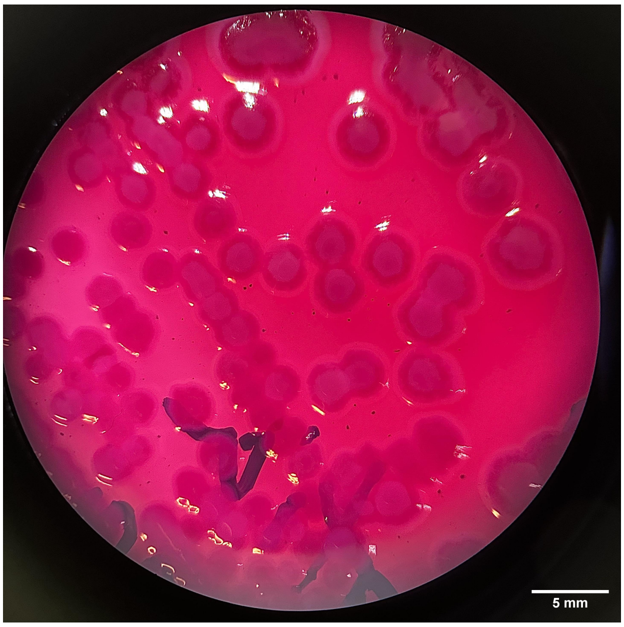


**Figure S2.** Morphological assessment using the MS-7-ZOOM stereoscopic trinocular microscope. Microscope magnification: 0.67-4.5x.

**Table S1.** Disk diffusion method results. Disk content represented in $\mu$g per disk.

| Antibiotic | Concentration, $\mu$g per disk | Zone diameter, mm | Results |
|---|---|---|---|
| Fosfomycin | 200 $\mu$g | 28 mm | Sensitive |
| Norfloxacin | 10 $\mu$g | 20 mm | Resistant |
| Erythromycin | 15 $\mu$g | 0 mm | Resistant |

*Section* S3. *Antibiotics resistance*

Extended data of the *Serratia rubidaea* 151 resistance assessment to a range of drugs presents all tested antibiotics in all tested concentrations and can be found in Tables S1 and S2, S3. The studied strain was susceptible to concentration values that had an MIC sign, and every value above it within the same column.

**Table S2.** Antimicrobial susceptibility tested panels for the studied strain (Part 1). Concentrations are mentioned in $\mu$g/mL.

| Ampicillin | Ampicillin/ sulbactam | Cefazolin | Cefuroxime | Aztreonam | Gentamicin | Amikacin | Colistin | Trimethoprim/ sulfamethoxazole | Cipro- floxacin | Chloro- amphenicol | Tetracycline |
|---|---|---|---|---|---|---|---|---|---|---|---|
| 128 | 128/64 | 16 | 64 | 16 | 32 | 64 | 16 | 4/76 | 8 | 32 | 32 |
| 64 | 64/32 | 8 | 32 | 8 | 16 (MIC) | 32 | 8 | 2/38 | 4 | 16 | 16 (MIC) |
| 32 | 32/16 | 4 | 16 | 4 | 8 | 16 | 4 | 1/19 (MIC) | 2 | 8 | 8 |
| 16 | 16/8 | 2 | 8 | 2 | 4 | 8 | 2 | 0.5/9.5 | 1 | 4 | 4 |
| 8 | 8/4 | 1 | 4 | 1 | 2 | 4 (MIC) | 1 | 0.25/4.75 | 0.5 | 2 | 2 |
| 4 | 4/2 | 0.5 | 2 | 0.5 | 1 | 2 | 0.5 | 0.12/2.38 | 0.25 | 1 | 1 |
| 2 | 2/1 | 0.25 | 1 | 0.25 | 0.5 | 1 | 0.25 | 0.06/1.19 | 0.12 | 0.5 | 0.5 |
| 1 | 1/0.5 | 0.12 | 0.5 | 0.12 | 0.25 | 0.5 | 0.12 | 0.03/0.6 | 0.06 | 0.25 | K |

**Table S3.** Antimicrobial susceptibility tested panels for the studied strain (Part 2). Concentrations are mentioned in $\mu$g/mL.

| Piperacillin | Piperacillin/ tazobactam | Cefotaxime | Ceftazidime | Cefoperazone | Cefoperazone/ sulbactam | Cefepime | Meropenem | Ertapenem | Tigecycline | Netilmicin | Tobramycin |
|---|---|---|---|---|---|---|---|---|---|---|---|
| 128 | 128/4 | 8 | 16 | 64 | 64/32 | 16 | 16 | 2 | 8 | 16 (MIC) | 8 |
| 64 | 64/4 | 4 | 8 | 32 | 32/16 | 8 | 8 | 1 | 4 | 8 | 4 |
| 32 | 32/4 | 2 | 4 | 16 | 16/8 | 4 | 4 | 0.5 | 2 | 4 | 2 |
| 16 | 16/4 | 1 | 2 | 8 | 8/4 | 2 | 2 | 0.25 | 1 (MIC) | 2 | 1 |
| 8 | 8/4 | 0.5 | 1 | 4 | 4/2 | 1 | 1 | 0.12 | 0.5 | 1 | 0.5 |
| 4 | 4/4 | 0.25 | 0.5 | 2 | 2/1 | 0.5 | 0.5 | 0.06 | 0.25 | 0.5 | 0.25 |
| 2 | 2/4 | 0.12 | 0.25 | 1 | 1/0.5 | 0.25 | 0.25 | 0.03 | 0.12 | 0.25 | 0.12 |
| 1 | 1/4 | 0.06 | 0.12 | 0.5 | 0.5/0.25 | 0.12 | 0.12 | 0.015 | 0.06 | 0.12 | 0 |
